# Faster nitrogen cycling and more fungal and root biomass in cold ecosystems under experimental warming: a meta‐analysis

**DOI:** 10.1002/ecy.2938

**Published:** 2019-12-31

**Authors:** Alejandro Salazar, Kathrin Rousk, Ingibjörg S. Jónsdóttir, Jean‐Philippe Bellenger, Ólafur S. Andrésson

**Affiliations:** ^1^ Faculty of Life and Environmental Sciences University of Iceland Sturlugata 7 101 Reykjavík Iceland; ^2^ Department of Biology Terrestrial Ecology Section University of Copenhagen Universitetsparken 15 2100 Copenhagen Denmark; ^3^ Center for Permafrost (CENPERM) University of Copenhagen Øster Voldgade 10 1350 Copenhagen Denmark; ^4^ Centre Seve Department of Chemistry Faculty of Sciences Universite de Sherbrooke J1K2R1 Sherbrooke Quebec Canada

**Keywords:** belowground biomass, cold biome, enzyme activity, gene pool, meta‐analysis, N flux, N pool, warming

## Abstract

Warming can alter the biogeochemistry and ecology of soils. These alterations can be particularly large in high northern latitude ecosystems, which are experiencing the most intense warming globally. In this meta‐analysis, we investigated global trends in how experimental warming is altering the biogeochemistry of the most common limiting nutrient for biological processes in cold ecosystems of high northern latitudes (>50°): nitrogen (N). For comparison, we also analyzed cold ecosystems at intermediate and high southern latitudes. In addition, we examined N‐relevant genes and enzymes, and the abundance of belowground organisms. Together, our findings suggest that warming in cold ecosystems increases N mineralization rates and N_2_O emissions and does not affect N fixation, at least not in a consistent way across biomes and conditions. Changes in belowground N fluxes caused by warming lead to an accumulation of N in the forms of dissolved organic and root N. These changes seem to be more closely linked to increases in enzyme activity that target relatively labile N sources, than to changes in the abundance of N‐relevant genes (e.g., *amoA* and *nosZ*). Finally, our analysis suggests that warming in cold ecosystems leads to an increase in plant roots, fungi, and (likely in an indirect way) fungivores, and does not affect the abundance of archaea, bacteria, or bacterivores. In summary, our findings highlight global trends in the ways warming is altering the biogeochemistry and ecology of soils in cold ecosystems, and provide information that can be valuable for prediction of changes and for management of such ecosystems.

## Introduction

Over the past 50 yr, temperatures at high northern latitudes have increased more than twice that of the rest of the globe (IPCC 2018). Our understanding of the ecological consequences of such warming has been improved by meta‐analyses that synthesize observations across large spatial and temporal scales. Most of these efforts have been focused on aboveground processes (e.g., Arft et al. 1999, Van Wijk et al. 2004, Elmendorf et al. 2012). Comparatively, we have a poor understanding of how warming is affecting belowground processes, in particular those related to nitrogen (N) cycling.

Nitrogen is the primary nutrient limiting net primary productivity in high‐latitude ecosystems (LeBauer and Treseder 2008, Wang et al. 2010, Kuypers et al. 2018). In these ecosystems, a large fraction of “new” N comes from diazotrophs (N‐fixing bacteria, free‐living or associated with soil crusts, mosses, or lichens) fixing atmospheric N_2_ into ammonia (Dickson 2000, Hobara et al. 2006, Rousk et al. 2016b). In these ecosystems, N fixation can be as high as 10 kg·ha^−1^·yr^−1^ (Cleveland et al, 1999, Gavazov et al. 2010, Rousk et al. 2015). Another source of “new” N is atmospheric N deposition, but in high‐latitude ecosystems such as the arctic and subarctic this input is low (<2 kg·ha^−1^·yr^−1^; Peñuelas et al. 2013). When biomass decomposes, N‐rich organic molecules such as amino acids and peptides are released. This organic N can be taken up by plants and microbes (Chapin et al. 1993, Kielland 1994, Schimel and Chapin 1996), or be further mineralized releasing ammonia. Ammonia can either (1) be taken up by plants or microbes, or (2) be oxidized by microbes to nitrate (${\text{NO}}_{3}^{-}$), a more mobile form of N that is also accessible to plants and microbes (Liu et al. 2018). Thus, belowground N is either incorporated into living plant roots or microbial biomass (or soil organic matter, SOM), or it is dissolved as organic matter (DON; e.g., amino acids) or as inorganic ions (DIN), primarily as ammonium ions and nitrate. From now on, we will refer to these N pools as (1) microbial biomass N (MBN), (2) root N content, (3) DON, (4) ammonia, and (5) nitrate, and to the sum of these pools as total soil N (6).

The rate at which N moves between pools (i.e., N fluxes) is sensitive to temperature (Bai et al. 2013, Zhang et al. 2015), suggesting that intense warming at high latitudes can alter the biogeochemistry of belowground N in these ecosystems. At a global scale, the largest N fluxes belowground are uptake of inorganic ions (${\text{NH}}_{4}^{+}$ and ${\text{NO}}_{3}^{-}$) by roots or microbes, ammonification (production of ${\text{NH}}_{4}^{+}$ from organic N), nitrification (oxidation of ${\text{NH}}_{4}^{+}$ to ${\text{NO}}_{3}^{-}$) and denitrification (reduction of ${\text{NO}}_{3}^{-}$ to N_2_O or N_2_; Kuypers et al. 2018). Increased temperatures can have both direct and indirect effects on the fluxes of belowground N. Directly, warming can alter the rates of enzyme‐driven processes (e.g., proteases; Zhang et al. 2015). Indirectly, it can alter fluxes via changes in soil moisture (Rousk et al. 2018), in the composition and/or structure of microbial communities (Chen et al. 2015), in litter quality (Rinnan et al. 2008), or in essential nutrient availability (i.e., metal cofactors) supporting key enzymatic processes (Li et al. 2014). Either way, warming‐induced changes in the fluxes of N belowground could release or intensify N limitation for biological processes. This may have transformative consequences in cold ecosystems, where organisms are adapted to low N availability (Wang et al. 2010).

In this meta‐analysis, we asked if and how experimental warming alters pools and fluxes of belowground N in cold, high northern latitude ecosystems. To estimate the effects of warming on cold‐adapted ecosystems, regardless of their location, and to account for differences across latitudes, we also included experiments from intermediate and high southern latitudes. We hypothesized that warming accelerates N fluxes belowground, increasing the abundance of accessible N for plants and microbes, and ultimately accelerating N uptake and biomass growth. We expect this meta‐analysis to highlight trends in the ways warming affects belowground N cycling in cold ecosystems across the globe.

## Materials and Methods

We meta‐analyzed data from 94 studies: 93 published in peer‐reviewed journals and 1 unpublished (details below). When studies included sites in more than one biome (e.g., Brzostek et al. 2012), we analyzed results from each biome separately. This led to a total sample size of 100 data sets (Metadata S1 and Data S1). Sites were located primarily at high latitudes (>50° N, 60/100; >50° S 3/100), but for comparison we also included in our analysis high‐elevation grasslands and tundra, as well as boreal and temperate forests at intermediate latitudes (37/100).

### Data sets

We searched the published literature for manuscripts reporting on the experimental manipulation of temperature in cold biomes. We searched peer‐reviewed journal articles using the combination of terms: (“field” AND “warming” AND “nitrogen”) AND (“soil” OR “below ground” OR “belowground”) in Google Scholar. To do a more refined search for N pools, like microbial biomass N, and fluxes, like N fixation, we added terms like “AND (“microbial biomass nitrogen” OR “microbial biomass N”)” (2,330 results) and “AND (“nitrogen fixation” OR “N fixation”)” (17,500 results), respectively. Similarly, for a more refined search for enzyme activity, composition/structure of microbial communities, and abundance of genes relevant for N cycling, we added the terms “AND enzyme” (32,100 results); “(AND microbial AND (community OR structure))” (85,800 results); or (AND “gene” AND “amoA” OR “nirS” OR “nirK” OR “nosZ” OR “nifH”) (3,090 results). After each search, we exhaustively screened out studies that (1) did not include a field warming experiment (i.e., we did not analyze data from laboratory or modeling experiments); or (2) were not located in “cold regions,” arbitrarily defined here as sites with mean annual temperature (MAT) equal or lower than 5°C; or (3) did not have data about soil N responses to warming (see complete list of response variables and references in Metadata S1 and Data S1). In total, we found 93 studies that met our criteria. Publications after July 2018 or not written in English were excluded. The only exception was Gong et al. (2019), which was included in our analysis because it contributed with data for N_2_O emissions (from unfertilized plots only), the N flux with the widest confidence interval (CI) range and the otherwise lowest sample size (see results).

In addition to data from peer‐reviewed journal articles, we analyzed data from a warming experiment in a dwarf shrub heath at Audkuluheidi, Iceland (65°16′ N, 20°15′ W; I. S. Jónsdóttir and R. Guicharnaud, *unpublished data*). Information about this site can be found in Jónsdóttir et al. (2005).

### Data collection

We collected mean ($\bar{x}$), standard deviation (*s*), and sample size (*n*) data from warming (subscript *w*) and control (subscript *c*) treatments. We calculated *s* based on standard error and *n* when needed. When data were available only in figures, we used data‐extraction software (Plot Digitizer 2.6.8, http://www.plotdigitizer.sourceforge.net) to extract numeric data.

We collected data for three N fluxes: N fixation, N mineralization (both as a single variable and differentiated between ammonification and nitrification), and N_2_O emissions; and for six N pools: microbial biomass N, root N, DON, ammonia, nitrate, and total soil N. We also collected data for six N‐relevant enzymes: protease (catalyses the hydrolysis of proteins and peptides), urease (catalyses the hydrolytic release of urea to ammonia), leucine aminopeptidase (LAP; catalyses the hydrolysis of leucine residues at the N‐terminus of proteins and peptides), N‐acetylglucosaminidase (NAG; catalyses the hydrolysis β‐N‐acetylglucosamine residues from oligosaccharides), and phenol oxidase and peroxidase (PO and POX; depolymerize lignin and other complex compounds); as well as for six N‐relevant genes: bacterial and archeal *amoA* (encode for ammonia monooxygenase), *nirS* (encoding cytochrome cd1 nitrite reductase), *nirK* (encoding copper‐containing nitrite reductase), *nosZ* (encoding nitrous oxide reductase), and *nifH* (encoding nitrogenase iron protein). Finally, to analyze possible relationships between belowground N cycling and soil ecology, we collected data of abundance and/or biomass of microbes, microfauna (i.e., fungivores and bacterivores) and plant roots.

### Data classification

We classified data based on soil depth, latitude, and biomes. Soil depths were classified as 5, 10, 15, and below 15 cm. We made approximations when needed. For example, soil depths of 2.1 ± 0.5, 4.5 ± 1.3, 10.3 ± 2.1, and 10.6 ± 2.1 cm in Björk et al. (2007), were approximated to 5, 5, 10, and 10 cm, respectively (details in Metadata S1 and Data S1). In some cases, information was limited and it was not possible to classify soil depth within these categories. These studies were excluded from comparisons between depths, but were included in the rest of the analyses.

**Figure 1:**
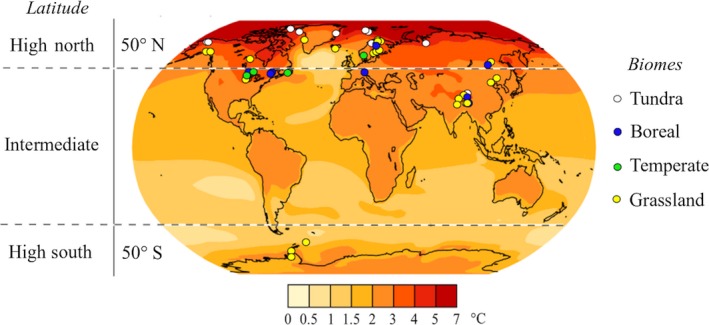
Projections of global surface temperatures for 2100 (mean temperature 2080–2100 to 1980–2000, January–December AR5 CMIP subset) and locations of field studies included in this meta‐analysis (white circles). Warming projections are based on the IPCC Fifth Assessment Report Climate Change Atlas (RCP 4.5) and were generated using the Climate Explorer Tool (https://climexp.knmi.nl/plot_atlas_form.py). The location of the studies on the global map was generated using the *borders* function from the R package *maps*. The sites in Asia are over 3,000 meters above sea level. Biomes were classified based on MAT and MAP (Appendix S1: Fig. S1).

Limits for high northern, intermediate, and high southern latitudes were arbitrarily set at above 50° N, between 50° N and 50° S, and above 50° S, respectively (Fig. 1). The 50° N threshold was arbitrarily selected to delimit the region of the Earth that is projected to continue experiencing the most intense warming (Fig. 1).

Biomes were classified as tundra, boreal, temperate, and grassland based on MAT and mean annual precipitation (MAP), as in Woodward et al. (2004) (Appendix S1: Fig. S1). In most cases, biome classification using this approach matches the ecosystem type described in previous articles (e.g., Biasi et al. 2008, Rossi et al. 2013, Sistla et al. 2013), but in some cases it does not. For example, the study site in Allison and Tresseder (2008) is a boreal forest, but based on MAT (−2°C) and MAP (303 mm) and the method proposed by Woodward et al. (2004), this site is classified as a grassland (Appendix S1: Fig. S1). Here we decided to be consistent in the use of MAT and MAP to classify biomes and recommend readers to take into account the limitations of this approach when interpreting our results. Finally, because all the reviewed articles reported vegetation cover, when MAP was below ca. 260 mm (i.e., desert), we classified biomes as tundra (MAT < −5°C) or grassland (MAT > −5°C; Appendix S1: Fig. S1).

### Data analysis

We conducted a meta‐analysis using standardized mean differences as a measure of effect size (Gleser and Olkin 2009). We calculated standardized mean differences (*y*) and corresponding sampling variances (*v*) as

(1) $$y\;=\;\frac{{\bar{x}}_{w}-{\bar{x}}_{c}}{S_{p}}$$

(2) $$v\;=\;\frac{1}{n_{w}}\;+\;\frac{1}{n_{c}}\;+\;\frac{y^{2}}{\left(2*n\right)}$$

where *S_p_*, the pooled standard deviation, is computed as:

(3) $$S_{p}\;=\;\sqrt{\frac{\left(\left(n_{w}-1\right)*s_{w}^{2}\right)\;+\;\left(\left(n_{c}-1\right)*s_{c}^{2}\right)}{\left(n_{w}\;+\;n_{c}-2\right)}}$$

Calculating multiple *S_p_* from the same study, based on the repeated use of data from the control treatment, can lead to autocorrelation (Gleser and Olkin 2009). To account for this potential correlation, we constructed a variance–covariance matrix of the effect size estimates (Gleser and Olkin 2009).

To determine whether the warming treatments had a significant effect on the response variables, we employed a fixed‐effect model using the statistical package *metafor* (Viechtbauer 2010) in R (version 3.4.2). When there were more than 10 studies for a response, we tested whether the effects of warming on that response differed across soil depths, latitudes, and biomes. The number of studies for N fixation and N_2_O emissions was lower than 10 (see results). But given the emphasis of our discussion on N cycling, we tested the effects of warming on these variables across soil depths, latitudes, and biomes as well.

When the number of studies for a particular analysis was less than 15, we generated 95% confidence intervals (CI) using bootstrapping (10,000 iterations). We used the bootstrapping bias‐corrected and accelerated (BCa) method, which was designed to perform reasonably well across a wide range of scenarios (Puth et al. 2015). We considered the effect of warming on response variables to be significant when CI did not overlap with zero. When the number of studies for a particular analysis was one, we show the calculated effect size but could not calculate CIs and therefore could not conclude about its statistical significance, or lack thereof.

To determine whether the effects of warming on response variables were dependent on the magnitude of the warming (ranging from ca. 0 up to 4°C; Metadata S1 and Data S1) and/or the length of the experimental manipulation (ranging from a few weeks up to 22 yr; Metadata S1 and Data S1), we conducted a meta‐regression analysis (*rma* function, *metafor* package; Viechtbauer 2010). To determine whether the effects of climate manipulations were caused by warming and/or by (unintentional) changes in soil moisture, we also analyzed the effects of moisture on response variables. In addition to analyzing the independent effects of warming, length of experiment, and moisture on response variables, we analyzed their two‐level interactions. Response variables were meta‐regressed one at a time.

Notice that methodological differences between the meta‐regression and the meta‐analysis based on standardized mean differences could lead to different and even apparently contradictory results. For example, soil temperatures ranged from ca. −5°C (−6.2 ± 0.5°C and −4.2 ± 0.5°C in control and warmed plots in Natali et al. 2012) to ca. 23°C (22.4 ± 0.5°C and 23.5 ± 0.9°C in 1‐yr control and warmed plots in Zhang et al. 2011), across data sets in this study. A single response variable may not significantly respond to warming treatment as assessed in our meta‐analysis, despite that it may vary significantly with temperature across sites and sampling times, which would be revealed by our meta‐regression. Also, not all studies report soil temperature and moisture data, which is needed for the meta‐regression but not for the analysis of standardized mean differences (except for the analysis of soil temperature and moisture). Therefore, the sample sizes for both approaches were slightly different, and this could also affect our results.

Finally, although in some cases all the studies that report measurements for one single variable used the same method (e.g., the chloroform‐fumigation extraction method [Brookes et al., 1985] for estimating microbial biomass N), in other cases we aggregated different methods (e.g., Sistla et al. 2013 reports abundance of fungivores and bacterivores in micrograms C per gram of soil, whereas Thakur et al. 2016 report these values in number of individuals per 20 g of fresh soil), which warrants caution in interpretation. Analyzing differences between methods (e.g., as in Rustad et al. 2001) is beyond the scope of this study, but we provide a data set (Metadata S1 and Data S1) that would be useful for such an endeavor.

## Results

### Effects of experimental warming on soil temperature, moisture, and pH

**Figure 2:**
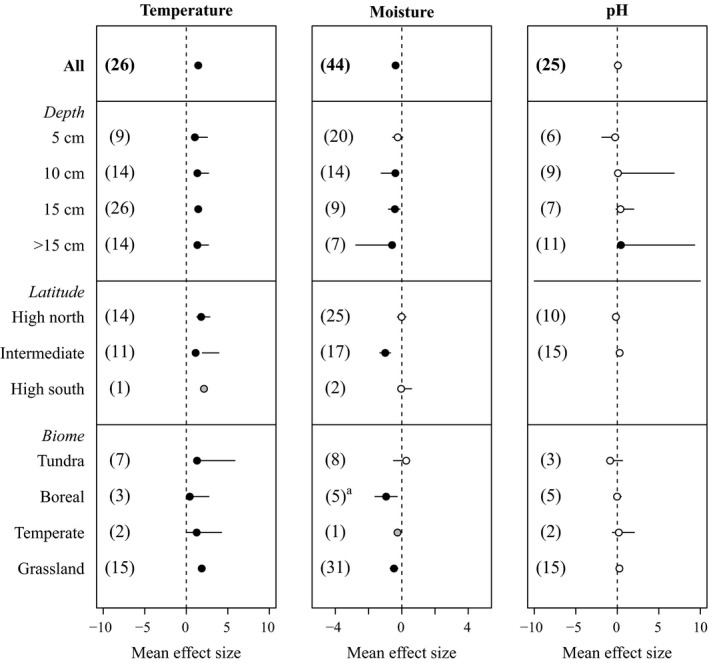
Effects of experimental warming on soil temperature, moisture, and pH in cold ecosystems. Numbers in parentheses indicate the number of studies. Solid and open symbols indicate statistical significance (i.e., bars showing 95% confidence interval [CI] do not overlap with zero) and no significance, respectively. Gray symbols (without CI) are used when there is only one study. Note scale differences of *x* axes. ^a^BCa bootstrapped CI: (−35.297, −0.707). For simplicity, in the figure we show the non‐bootstrapped CI (−1.615, −0.272), which also indicates statistical significance.

As expected, experimental warming in cold ecosystems has increased soil temperatures (CIs: 1.144, 1.734) across biomes, latitudes and soil depths (Fig. 2). Warming has decreased moisture content (CIs: −0.571, −0.173) below 5‐cm depth, at intermediate latitudes, in grasslands and boreal ecosystems (Fig. 2). In contrast to temperature and moisture, pH is largely unresponsive to experimental warming (Fig. 2). Taken together, our analysis suggests that experimental warming in cold ecosystems has increased soil temperatures, has decreased moisture, and has not affected soil pH.

### Soil nitrogen pools and fluxes

**Figure 3:**
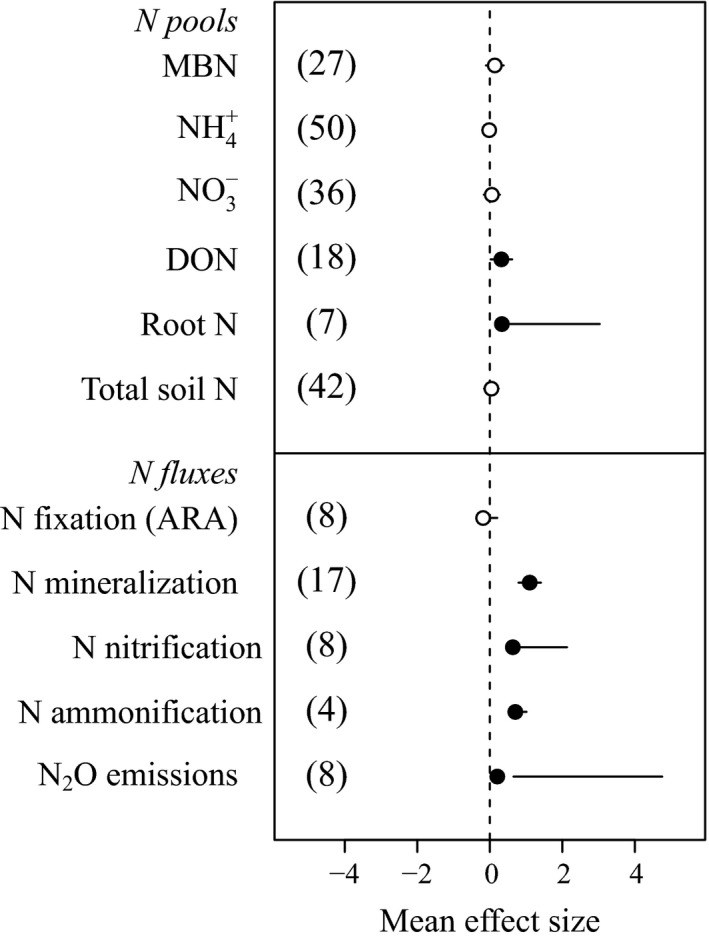
Effects of experimental warming on belowground N pools (more details in Appendix S1: Fig. S2) and fluxes (more details in Appendix S1: Fig. S3) in cold ecosystems. Root N indicates net N content, not concentration. Solid and open symbols indicate statistical significance (i.e., bars showing 95% confidence interval [CI] do not overlap with zero) and no significance, respectively.

Warming alters the biogeochemistry of N in soils of cold ecosystems. Overall, warming does not affect the pools of ammonia or nitrate, no matter the magnitude or length of the warming treatment (Fig. 3 and Appendix S1: Table S1). Aside from a moisture‐dependent, positive relationship between the magnitude of warming and microbial biomass N (Appendix S1: Table S1), and from the positive effect of warming in microbial biomass N in tundra soils (Appendix S1: Fig. S2), the microbial biomass N pool is generally unaffected by warming in cold ecosystems (Fig. 3). In contrast, warming in these ecosystems leads to an accumulation of DON (particularly in grasslands, Appendix S1: Fig. S2) and root N content (Fig. 3). The effect of warming on DON is marginally dependent on soil moisture (Appendix S1: Table S1). Interestingly, our meta‐analysis suggests that warming (within the conditions simulated by the studies included in this analysis) does not lead to a net depletion or accumulation of total soil N in cold ecosystems (Fig. 3).

In addition to altering the size of belowground N pools, warming in cold ecosystems alters N fluxes between pools. Both N mineralization (ammonification and nitrification) and N_2_O emissions increase with warming. Warming‐induced increases in N mineralization are consistent across soil depths, latitudes, and biomes (Appendix S1: Fig. S3). In contrast to N mineralization and N_2_O emission, we found no differences in N fixation rates between control and warmed plots across biomes and conditions (Appendix S1: Fig. S3). Our results highlight a dependence of N fixation on moisture, interacting with length of experiment (*P* = 0.039) and marginally with temperature (0.054) (Appendix S1: Table S1).

### Belowground living biomass

**Figure 4:**
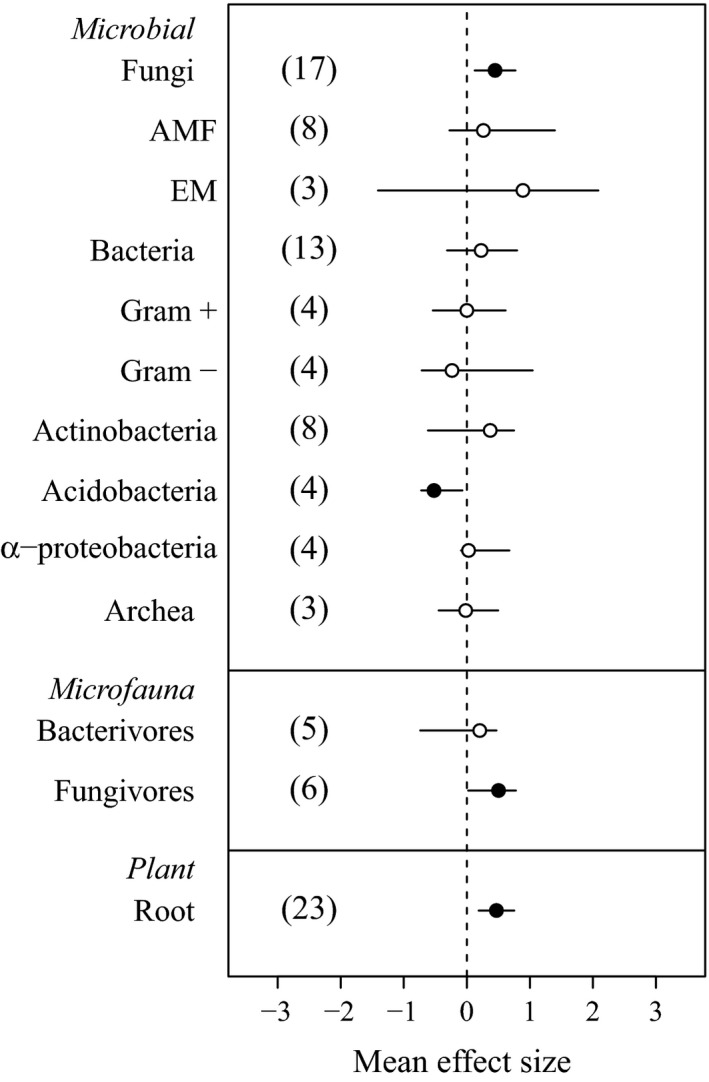
Effects of experimental warming on belowground living biomass (or abundance). AMF: arbuscular mycorrhiza; EM: ectomycorrhizae. Solid and open symbols indicate statistical significance (i.e., bars showing 95% confidence intervals [CI] do not overlap with zero) and no significance, respectively.

Warming in cold ecosystems increases the biomass and/or abundance of belowground fungi, fungivores, and plant roots (Fig. 4). Fungal biomass increases with warming below the soil surface, at high northern latitudes, and in grasslands (Appendix S1: Fig. S4). Root biomass responses to warming, which are significant at high northern and intermediate latitudes and in tundra and grassland ecosystems, do not show a clear pattern across soil depths (Appendix S1: Fig. S4). Our meta‐regression analysis suggests a positive relationship between bacterial biomass and temperature (alone and interacting with length of experiment; Appendix S1: Table S1), and a marginal relationship with moisture (alone and interacting with temperature; Appendix S1: Table S1). However, except for a negative effect on Acidobacteria (Fig. 4), we found no evidence of experimental warming across cold ecosystems affecting net bacterial, or archaeal, biomass.

### Enzyme activity

**Figure 5:**
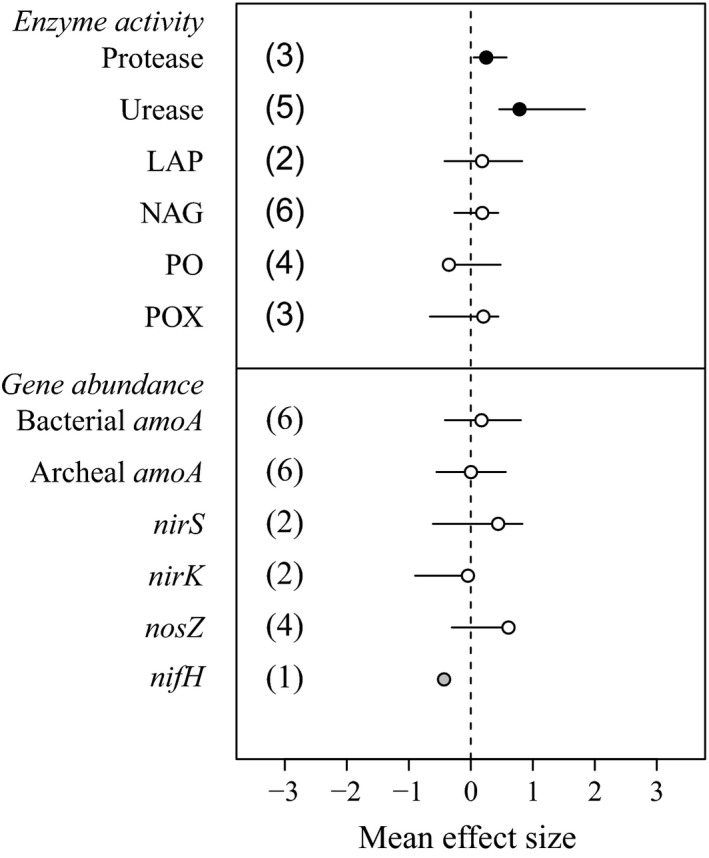
Effects of experimental warming on enzyme activity and on the abundance of genes relevant for N cycling belowground. LAP: leucine aminopeptidase, NAG: N‐acetylglucosaminidase, PO: phenol oxidase, and POC: peroxidase. Solid and open symbols indicate statistical significance (i.e., bars showing 95% confidence intervals [CI] do not overlap with zero) and no significance, respectively. Gray symbols (without CI) are used when there is only one study.

Relative to the data available for soil N pools and fluxes and for components of biomass in soils, there are few data on the effects of warming on enzyme activity and on abundance of N‐relevant genes in cold ecosystems. We recommend caution when interpreting results from analyses with a particularly low number of studies (e.g., N < 5), as it is the case for the enzymes protease, LAP, PO, and POX; and for the genes *nirS*, *nirK*, *nosZ*, and *nifH* (Fig. 5).

Considering the studies that met our search criteria, our analysis suggests that warming in cold ecosystems increases the activity of proteases and urease, but not of leucine aminopeptidase (LAP), N‐acetylglucosaminidase (NAG), phenol oxidase (PO), and peroxidase (POX; Fig. 5). Interestingly, the two responsive enzymes target relatively labile N sources such as urea and peptides, whereas three out of the four unresponsive enzymes (NAG, PO, and POX) target more recalcitrant N sources, such as polysaccharides and chitin metabolites.

### Abundance of N‐relevant genes

We found no evidence of warming altering the abundance of N‐relevant genes in a consistent way across cold ecosystems (Fig. 5). Here, we also recommend caution when interpreting results from analyses with a particularly low number of studies, as it is the case for *nirS*, *nirK*, *nosZ*, and *nifH*.

## Discussion

**Figure 6:**
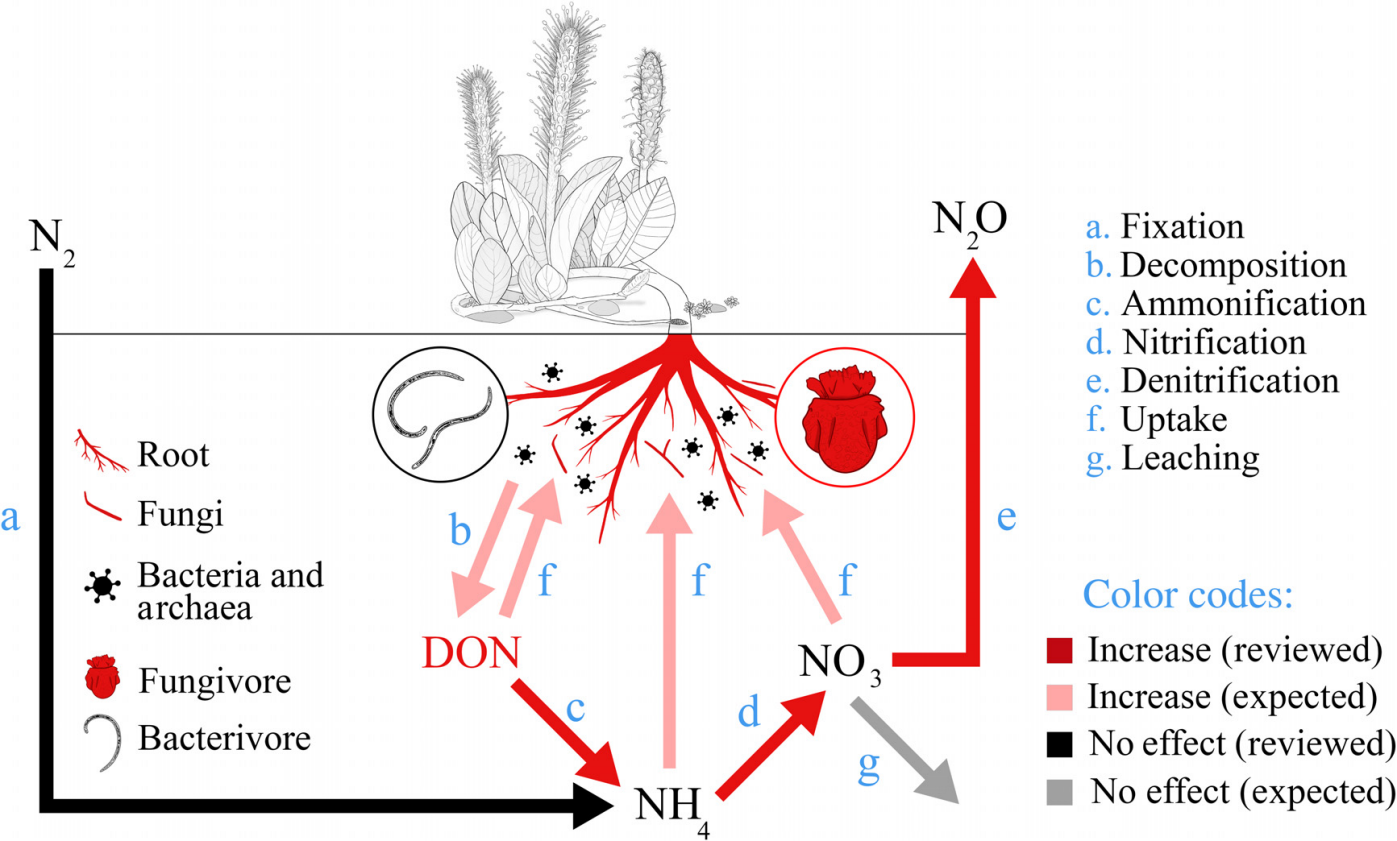
Conceptual representation of warming effects on N cycling and belowground communities in cold ecosystems. Fungivore is shown in red, indicating a positive effect of warming on fungivore abundance or biomass, but not necessarily on fungivore biomass N (not reviewed).

Our analysis highlights global responses of belowground N to warming in cold ecosystems. Overall, warming in cold ecosystems does not affect N fixation rates or the pools of inorganic N in soils. Also, warming in these ecosystems does not affect the abundances of bacteria and archaea, nor the abundance of genes relevant for N cycling (Fig. 6). However, warming in cold ecosystems accelerates N mineralization and N_2_O emission rates and leads to an accumulation of DON and root N content. Also, it increases the activity of enzymes that target relatively labile N sources, and favors the growth of roots, fungi, and fungivores (Fig. 6).

### No evidence of warming affecting N fixation in a consistent way across cold ecosystems

Although N fixation may respond to warming on a regional scale, the direction and magnitude of these responses can vary among regions and/or conditions, resulting in a nonsignificant response on a global scale. For example, in a subarctic wet heath in northern Sweden, overall growing‐season N fixation by moss‐associated diazotrophs was about three times larger in plots that had been warmed with dome‐shaped plastic greenhouses for 10 yr than in unmanipulated plots (Sorensen and Michelsen 2011). But the magnitude of the warming‐induced increase differed between moss species and between months (Sorensen and Michelsen 2011). In the same area, 21 yr of warming had either no effect or a negative effect on N fixation in the mosses *Hylocomium splendens* and *Aulacomnium turgidum*, respectively (Sorensen et al. 2012). The negative effect was attributed to warming facilitating the growth of vascular plants in areas previously covered by bare soil (possibly crusted), mosses, and lichens (Sorensen et al. 2012). A more recent study at the same site (Rousk and Michelsen 2017), and a similar experiment in the high Arctic, Greenland (Rousk et al. 2018), found no effects of warming on N fixation. Although overall conclusions are difficult to draw because of different factors modulating the responses of N fixation to warming (e.g., moisture, moss and cyanobacterial species, and long‐term changes in aboveground cover), our meta‐analysis suggests that the combination of positive and negative responses across cold ecosystems could result in a nonsignificant net effect of warming on N fixation at global scale.

### Net warming‐caused increases in N_2_O emissions across cold ecosystems

The effects of warming on N_2_O emissions vary widely between regions and conditions. Overall, warming in cold ecosystems has either no effect (e.g., Hu et al. 2010, Lamb et al. 2011) or a positive effect (e.g., Chang et al. 2017, Shi et al. 2012, Cui et al. 2018) on N_2_O emissions. When positive, the magnitude of this effect varies widely across studies. For example, in an alpine meadow of the eastern Tibetan Plateau, a 1°C increase in soil temperature for one growing season increased N_2_O emissions by ca. 30% (Shi et al. 2012). In contrast, in a boreal peatland dominated by *Betula fruticosa*, a soil warming of 2°C for 1 yr increased N_2_O emissions by more than 300% (Cui et al. 2018). These responses can be modulated by factors that were not explicitly taken into account in this meta‐analysis, such as N fertilization (Gong et al. 2019). For example, warming increased N_2_O emissions in an unfertilized arctic peatland from −0.16 ± 0.12 nmol·m^−2^·s^−1^ (net sink) to almost zero. But in fertilized plots (which were not considered in our meta‐analysis), warming decreased N_2_O emissions from 1.45 ± 0.30 (net source) to 0.92 ± 0.20 nmol·m^−2^·s^−1^ (Gong et al. 2019). Together, these results suggest that in the absence of limiting factors, such as moisture or nutrients, global warming will likely increase N_2_O emissions across cold ecosystems, potentially leading to a positive climate feedback (as predicted by models, e.g., Xu‐Ri et al. 2012), and that the magnitude of these responses will vary widely among biomes and conditions.

### Increases in N mineralization rates without changes of inorganic N pools

Interestingly, our meta‐analysis suggests that, at the global scale, warming‐induced increases in N mineralization rates (generally measured via buried bag incubations; e.g., Jonasson et al. 1993) are not followed by changes in inorganic N belowground (measured in soil cores; e.g., Jonasson et al. 1993). Most of the inorganic N in these soils is ammonium ion. Nitrate is commonly below detection limits (e.g., Jonasson et al. 1993, Chapin et al. 1995, Nordin et al. 2004). Aside from a few cases in which warming has led to accumulation (e.g., Chapin et al. 1995, Rinnan et al. 2007, Allison and Tresseder 2008) or depletion (e.g., Xiong et al. 2016) of inorganic N, in most cases there is no overall effect (e.g., Jonasson et al. 1993, Allison et al. 2010, Stark et al. 2018). In particular, there seems to be no warming‐induced N depletion via nitrification of ammonia to the more mobile nitrate and further leaching (Joseph and Henry 2008). The finding that warming increases N mineralization rates and does not affect belowground inorganic N pools may be linked to the observed increases in N_2_O emissions (denitrification rates) and/or may indicate a rapid, possibly warming‐increased uptake of inorganic N into living biomass (as further discussed below).

### Warming leads to a net accumulation of DON in cold ecosystems

Although N mineralization, a process that transforms organic N into ammonia, is enhanced by warming in cold ecosystems, we found no evidence of warming depleting belowground DON. On the contrary, we found a net positive effect of warming on soil DON. For instance, 2 yr of experimental warming in a lichen‐rich dwarf shrub tundra in Siberia (Biasi et al. 2008), increased gross N mineralization rates from 0.23 ± 0.04 to 0.34 ± 0.03 g NH_4−_ N·m^−2^·d^−1^ (48% increase), and at the same time increased DON from 2.1 ± 0.3 to 4.9 ± 1.7 g/m^2^ (133% increase). Similar increases in DON with warming have been observed elsewhere (e.g., Kane et al. 2014, Schaeffer et al. 2013). Although there are exceptions to this trend (e.g., Rousk et al. 2018, Stark et al. 2018), our meta‐analysis suggests that warming in cold ecosystems could lead to a net accumulation of belowground N in the form of DON.

Given that DON is a product of the decomposition of living matter, and that warming generally enhances aboveground (Arft et al. 1999, Walker et al. 2006, Elmendorf et al. 2012) and belowground plant biomass (our results and Pregitzer et al. 2000), our finding of warming increasing belowground DON is reasonable. The observation that warming in cold ecosystems increases both N mineralization and soil DON suggests that increases in the rate at which DON is added to soil are generally larger than increases in the rate at which DON is mineralized to inorganic N.

### Warming increases root biomass and root N across cold ecosystems

In addition to the warming‐induced increases in soil DON, our analysis highlights a widespread positive effect of warming on root N content. Warming‐induced increases in root N content are generally accompanied by increases in root biomass. This suggests that the effects of warming on root N content in cold ecosystems are linked to root biomass rather than to root N concentration. For instance, a warming experiment in an alpine meadow on the Qinghai‐Tibetan Plateau (Chang et al. 2017), decreased root N concentration in the top 10 cm soil layer from 14.7 ± 0.5 to 10.7 ± 1.2 g/kg (27% decrease). In contrast, root biomass went from 890 ± 114 to 1576 ± 136 g/m^2^ (77% increase), and root N content from 13.1 ± 2.5 to 16.7 ± 0.8 g/m^2^ (27% increase). Although in this study root N concentration is not reported for deeper soil, both root biomass and root N content increased in depths ranging from 20 to 60 cm deep. In these depths, the difference in both biomass and root N content between control and warmed plots was ca. 100%. Observations from another study in the same region suggest that the effects of warming on root biomass can vary with the type of roots (i.e., fine or coarse) and ecosystem (i.e., plantation or natural forest; Li et al. 2015). Particularly, this study suggests that warming does not affect fine root biomass or N in plantations, or coarse root biomass or N in natural forests or plantations, but it increases fine root biomass and N in natural forests (Li et al. 2015). Although it is unclear how the effect of warming on root biomass and N varies with type of root and other factors, our observation of experimental warming increasing root biomass and N content across cold ecosystems suggests that plant roots from these ecosystems could act as a terrestrial N sink in a warmer world.

### Microbial biomass N unaffected by experimental warming across cold ecosystems

The responsiveness of root N to warming in cold ecosystems contrasts with the unresponsiveness of belowground microbial biomass N, which is consistent across soil depths, latitudes and biomes. Although at the local scale and under certain conditions warming can increase (e.g., Li et al. 2011, Ma et al. 2011, Sistla et al. 2013) or decrease (e.g., Fu et al. 2012, Weedon et al. 2012, Jing et al. 2014) microbial biomass N, in most cases it does not have any effect (e.g., Jonasson et al. 1999, Biasi et al. 2008, Zhou et al. 2013). Despite this unresponsiveness, the marginal positive relationship between the magnitude of experimental warming and microbial biomass N may reflect a temperature limitation of microbial biomass N (and likely microbial abundance; e.g., see Chen et al. 2015) in these cold ecosystems. In tundra, the biome with the lowest MAT (<−5°C) and therefore where temperature limitations are extreme, a warming such as the one projected for the coming decades (IPCC 2018), is enough to release this limitation and allow the accumulation of microbial biomass N.

The effect of warming on microbial biomass N can depend on factors that are not taken into account in this meta‐analysis. For instance, in a subarctic tundra, 19 yr of experimental warming increased microbial biomass N in plots without herbivory, but decreased it in plots with herbivory (Stark et al. 2018). Despite this caveat, and considering that our analysis covers a significant range of conditions, we conclude that warming, within the ranges projected for the coming decades, will likely only affect microbial biomass N of soils from tundra ecosystems. This does not necessarily imply that warming will not affect microbial activity in other cold ecosystems as well. In fact, the unresponsiveness of microbial biomass N and the responsiveness of microbially regulated processes, such as N mineralization and denitrification, suggests a decoupling between microbial biomass and function.

### Warming favors fungi (and fungivores) but not bacteria (or bacterivores) and archaea

Although warming does not have a net effect on microbial N in soils from most cold ecosystems, it does have an effect on the composition of soil microbial communities. In particular, we observed a trend of warming favoring fungi over bacteria and archaea. For example, in a subarctic tundra heath a ca. 1°C warming simulated with open‐top chambers (OTCs) for 3 yr increased fungal biomass from 23 ± 1 to 27 ± 0.7 nmol/g, without affecting bacterial biomass (Rousk et al. 2016a). Considering our observation of warming benefiting plant roots (see also Pregitzer et al. 2000), and the overwhelming amount of evidence for warming stimulating aboveground plant growth in cold ecosystems (e.g., Arft et al. 1999, Walker et al. 2006, Bjorkman et al. 2018), the positive effect of warming on soil fungal communities might be direct, indirect through a temperature‐dependent flux of C from plants to fungi (e.g., Hawkes et al. 2008), or a combination.

The lack of warming effects on microbial biomass N in most ecosystems, as well as on bacterial and archaea biomass and the positive effect on fungal biomass (but see Bouskill et al. 2014), may indicate not just a generalized growth response of fungi across cold ecosystems, but also a change in their stoichiometry. Fungi, especially fungal species capable of supplying host plants with N, proliferate in N‐poor ecosystems such as the Arctic and Subarctic (Hobbie and Hobbie 2008). In an Alaskan arctic tundra, it was estimated that 61–86% of the plant N was supplied by fungal symbionts, whereas plants invested 8–17% of their net primary production to support their fungal symbionts (Hobbie and Hobbie 2006). It is possible that the generalized positive effect of warming on fungal biomass and root N content, but not on microbial N, indicates a growth of C‐rich fungal structures that serve to supply N to their host plants rapidly. For example, warming could promote the growth of C‐rich, nutrient‐seeking hyphal networks, while suppressing the formation of N‐rich, nutrient‐storing vesicles (Hawkes et al. 2008), with a net increase in fungal biomass. Although a more comprehensive analysis would need to take into account factors that can affect fungal biogeochemistry that were not addressed in this analysis (e.g., N deposition; Lilleskov et al. 2002), it seems reasonable that as winters, especially in high latitude ecosystems, continue getting shorter and milder (IPCC 2018), a temperature‐driven change in the nutrient‐seeking versus nutrient‐storing strategy of soil fungal communities would affect their overall C&hairsp;:&hairsp;N balance.

Alternatively, warming could alter the composition of fungal communities in cold ecosystems (e.g., Deslippe et al. 2012). Ectomycorrhizae (EM) plants are more efficient in their use of N and have access to more complex N sources than arbuscular mycorrhiza (AMF) plants (Cornelissen et al. 2001). At the ecosystem level, EM‐dominated systems, which proliferate in high‐latitude and high‐altitude biomes (Read and Perez‐Moreno 2003), accumulate more carbon per unit of N than AMF‐dominated systems (Zhu et al. 2018). Also, and consistent with our previous analysis about nutrient‐seeking versus nutrient‐storing structures, EM communities can build more extensive mycelial networks than their AMF counterparts (Zhu et al. 2018). Although we did not find a consistent response of EM abundance to warming across cold ecosystems, one out of the three sites considered in this part of our analysis (i.e., tundra in Clemmensen et al. 2006) shows that warming in the Arctic can increase EM abundance. In this case, the positive effect of warming on EM was linked to the dominance of EM plants (*Betula nana*, Clemmensen et al. 2006). On the other hand, warming in cold ecosystems could affect fungal groups that because of a lack of data were not taken in account in this meta‐analysis, such as ericoid mycorrhiza and dark septate endophytes (Olsrud et al. 2010). Taken together, these observations suggest that an increase in fungal biomass, with no change in bacterial or archaeal biomass, and with no change in microbial biomass N, could be the result of warming favoring the growth of fungal species with different N use strategies. In particular, it could indicate a disproportionately positive effect on N‐use efficient fungal groups over less efficient ones.

Either way, our meta‐analysis suggests that warming in cold ecosystems stimulates the growth of roots and fungi, leading to an accumulation of N in increased root biomass but not in microbial biomass. We encourage the testing of these and/or complementary hypotheses.

Also, our meta‐analysis suggests that warming in cold ecosystems increases the abundance of fungivores. Although we did not find enough data to meta‐analyze factors influencing the effects of temperature on the abundance of fungivores and bacterivores, at local scale this effect can vary, for example, with soil depth (Sistla et al. 2013) and with the age of individuals (Alatalo et al. 2017). If the metabolic responses of bacterivores and fungivores to warming are similar, the observation of warming not affecting bacteria and bacterivores, and positively affecting fungi and fungivores, suggests that the increased numbers of fungivores are due to the increase in their food supply (i.e., fungi).

### Warming affects enzyme activity but not gene pools

Despite the clear effects of warming on belowground communities in cold ecosystems, we found no evidence of warming affecting the abundance of genes relevant to N cycling considered in this analysis. This is consistent with results from a cross‐continental study associated with the International Tundra Experiment (ITEX), that found no effects of experimental warming on the abundance of belowground pools of genes involved in inorganic N cycling (S. Hallin, *unpublished*). In contrast, we found that warming consistently increased the activity of some N‐relevant enzymes, particularly of enzymes that target relatively labile N sources, such as urea and peptides. Although the number of studies in this part of our analysis (both of gene abundance and enzyme activity) is lower than what is generally considered optimum for meta‐analyses (Puth et al. 2015), it is interesting that the trends observed are consistent across large spatial scales (e.g., the Arctic, Lamb et al. 2011; and the Antarctic, Yergeau et al. 2012). Taken together, these results suggest that the effects of warming on belowground N cycling in cold ecosystems are more closely linked to enzymatic salvage of N from DON than to fluxes of inorganic N catalyzed by the products of the genes *amoA*, *nirS*, *nirK*, *nosZ*, and *nifH*.

Enzymatic responses to warming in cold ecosystems could be linked to the abundance of specific microbial groups. For example, EM produce more extracellular enzymes (including proteases and urease; Martin and Nehls 2009) to mine nutrients from SOM than AMF (Read and Perez‐Moreno 2003). Considering the positive effect of warming on the activity of proteases and ureases, and the positive effect of warming in two out of the three sites considered in our analysis for EM abundance, we cannot discard the possibility for the positive effect of warming on enzyme activities that target labile N sources to be linked to the abundance of their fungal producers.

The microbial enzymes that catalyze belowground cycling of inorganic N use metal cofactors, such as iron (Fe), copper (Cu), and molybdenum (Mo). The concentration and bioavailability of these and other metals in soil can change with temperature (Li et al. 2014). Warming in cold ecosystems can affect metal biogeochemistry in soil directly, for example, by increasing rates of microbial decomposition of soil organic matter, concomitant availability of heavy metals, and ultimately metal uptake by plants (Rajkumar et al. 2013); and/or indirectly, for example, by altering precipitation patterns. In the Arctic, temperature‐driven increases in precipitation and the higher contribution of rain to total precipitation (IPCC 2018), could lead to more intense weathering and release of trace metals from soil minerals. Considering that the availability of metal cofactors, such as Mo, can limit metal‐dependent processes, such as N fixation (Rousk et al. 2017, Jean et al. 2013), we may need to understand the effects of global warming on metal biogeochemistry to understand its effects on belowground N cycling fully.

## Conclusions

In summary, our meta‐analysis highlights global trends in the ways warming affects soil N cycling and belowground communities in cold ecosystems. In particular, our results show that field experiments simulating temperatures projected for the coming decades in cold ecosystems (IPCC 2018) consistently result in increased N mineralization and N_2_O emission rates; an accumulation of root and dissolved organic N; and net growth of roots, fungi, and fungivores. One of the limitations of this study is also its strength: although this paper aggregated 94 studies in total, only small subsets of studies overlap enough in their measured response variables to highlight large‐scale patterns with high statistical confidence. In this sense, our meta‐analysis provides a comprehensive view of the belowground N responses to warming across cold ecosystems and highlights the components of this soil N–climate system that remain more obscure.

## Supporting information

AppendixS1

DataS1

MetadataS1
