# Supplementary material for: Faster nitrogen cycling and more fungal and root biomass in cold ecosystems under experimental warming: a meta‐analysis
Source: Ecology. 2019 Dec 31;101(2):e02938. doi: 10.1002/ecy.2938 (PMC7027553; doi:10.1002/ecy.2938)
Supplement: Supplementary file 1 — AppendixS1 [file ECY-101-e02938-s001.pdf]

Supporting Information. Alejandro Salazar, Kathrin Rousk, Ingibjörg S. Jónsdóttir, Jean-Philippe Bellenger, and Ólafur S. Andrésson. 2019. Faster nitrogen cycling and more fungal and root biomass in cold ecosystems under experimental warming: a meta-analysis. *Ecology*.

Appendix S1

Supplementary figures

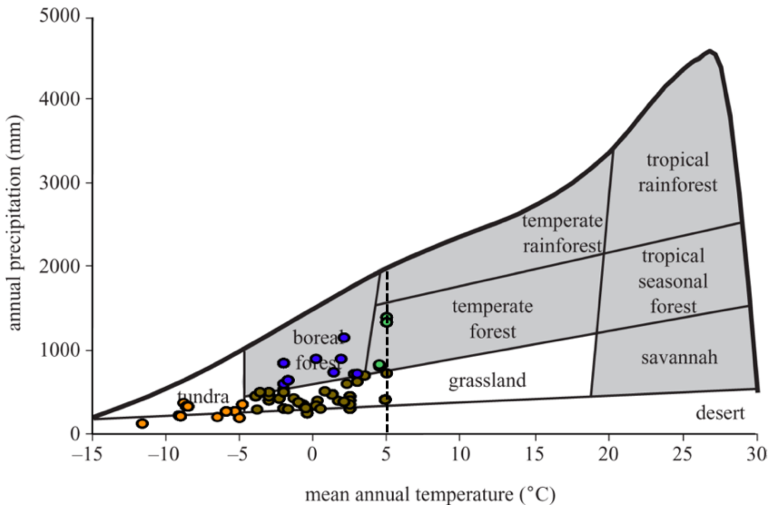

Figure S1. Biome classification based on MAT and MAP. The different colors of the symbols indicate when biomes were classified as tundra (orange), boreal (blue), temperate (green), and grassland (brown). One point can represent the location of multiple studies, and one study can have more than one point. Dashed line at MAT = 5 °C indicates our threshold for cold ecosystems in this meta-analysis. Studies where MAT was lower than -15 °C (not shown in figure; Lamb et al. 2011, Rossi et al. 2013) were classified as tundra. Modified with authorization from Woodward et al. (2004).

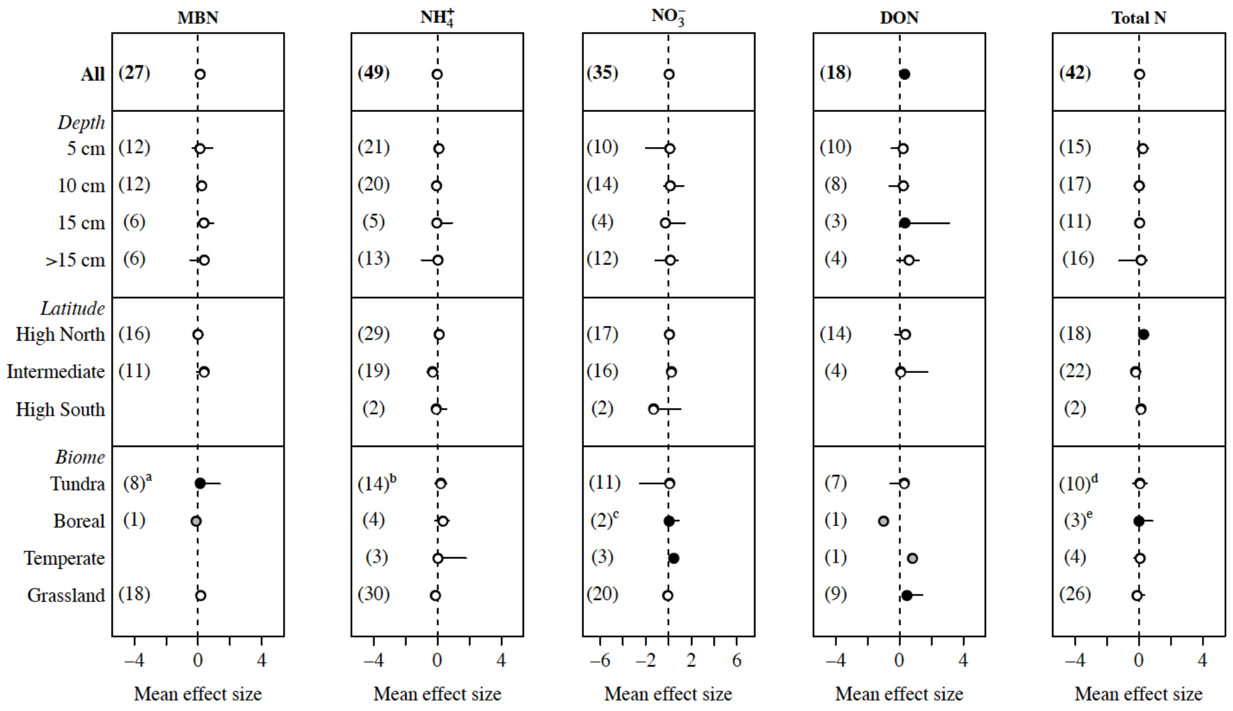

Figure S2. Effects of experimental warming on belowground N pools in cold ecosystems. MBN: microbial biomass N. DON: dissolved organic N. Solid and open symbols indicate statistical significance (i.e. bars showing 95% CI do not overlap with zero) and no significance, respectively. Grey symbols (without CI) are used when there is only one study. Note scale differences of x axes.

[a] Symbol overlaps with zero line, but mean effect size (0.128) and CI (0.207, 1.363) are both positive.

[b] Mean effect size (0.216) is outside the BCa bootstrapped CI (-0.906, -0.113). CI for this value in the figure (-0.135, 0.566) is not bootstrapped and, contrary to the bootstrapped CI, indicates no significance.

[c] Symbol overlaps with zero line, but mean effect size (0.068) and CI (0.068, 0.925) are both positive.

[d] BCa bootstrapped CI: (-7.962, 0.525). For simplicity, in the figure we show the non-bootstrapped CI (-0.360, 0.483), which also indicates no statistical significance.

[e] Symbol overlaps with zero line, but mean effect size (0.013) and CI (0.135, 0.854) are both positive.

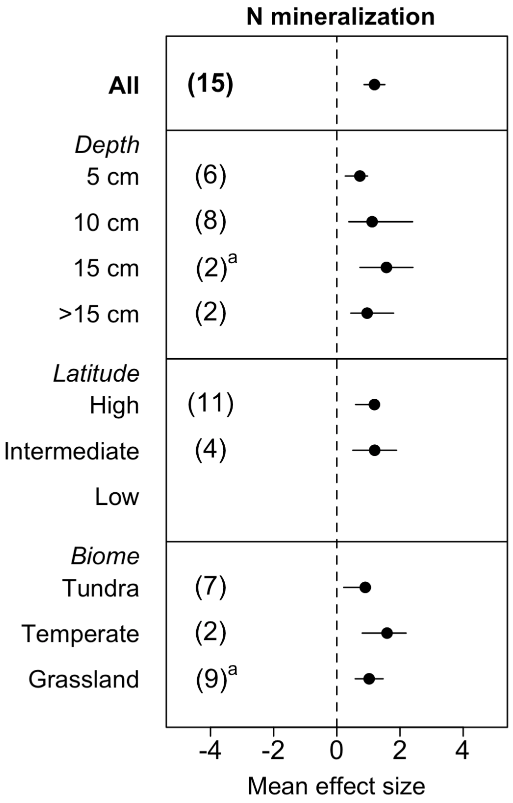

Figure S3. Effects of experimental warming on belowground N mineralization rates in cold ecosystems. No data found from arctic and boreal biomes. Solid symbols indicate statistical significance (i.e. bars showing 95% CI do not overlap with zero).

[a] Mean effect size for N mineralization values at 15 cm depth (1.571) and Grassland (1.023) are outside the BCa bootstrapped CIs (1.890, 2.226 and 1.290, 2.068; respectively). For simplicity, in the figure we show the non-bootstrapped CI (0.733, 2.408 and 0.582, 1.464; respectively), which also indicate statistical significance.

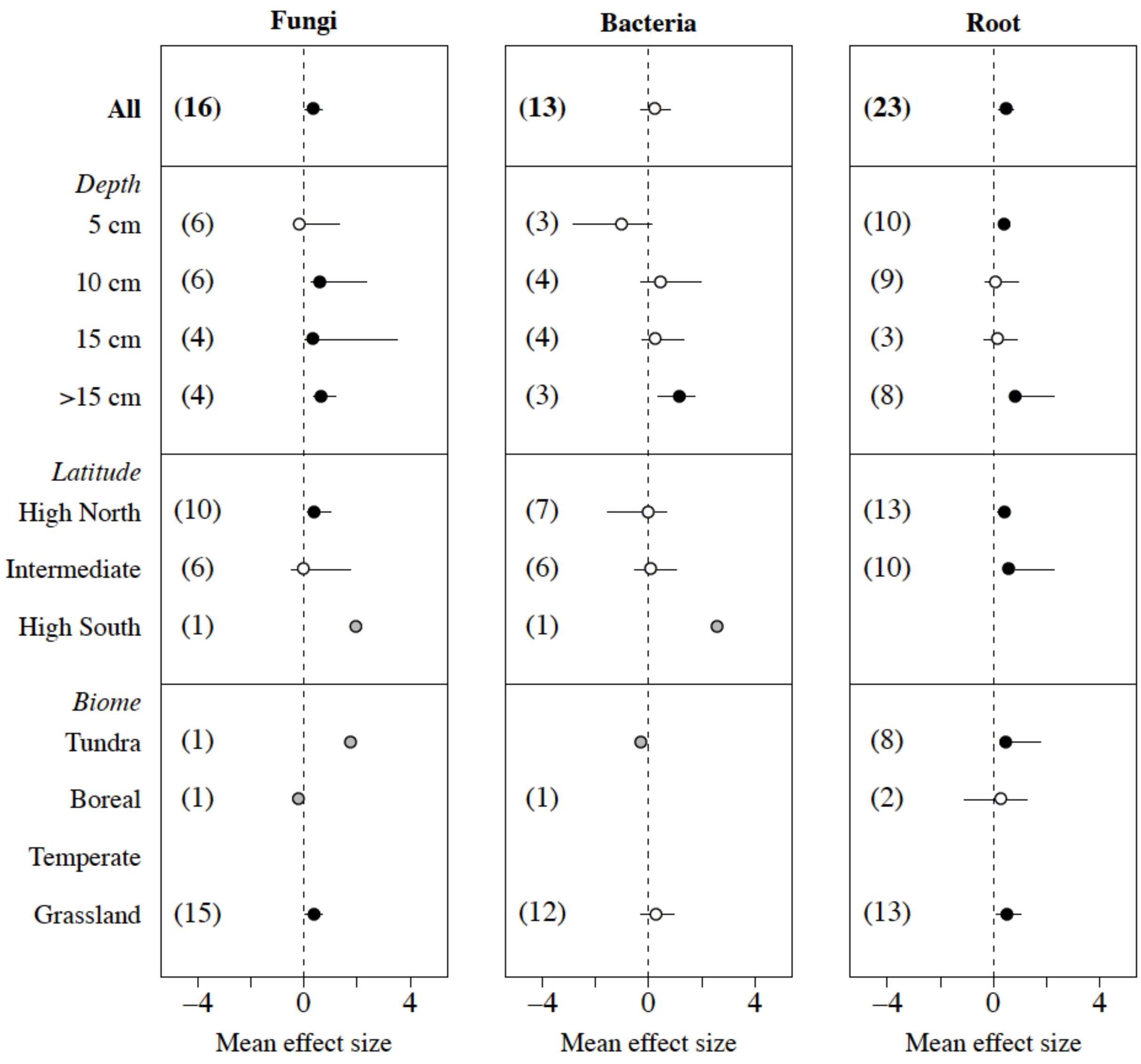

Figure S4. Effects of experimental warming on fungal, bacterial and root biomass (or abundance) in cold ecosystems. Solid and open symbols indicate statistical significance (i.e. bars showing 95% CI do not overlap with zero) and no significance, respectively. Grey symbols (without CI) are used when there is only one study.

Supplementary table

Table S1. Meta-regression results showing the relationships, or lack of, between response variables (i.e. effect sizes of microbial biomass N (MBN), ammonium, nitrate, DON, total N (TN), N mineralization (Nmin), N fixation (Nfix), fungi, bacteria and root biomass) and temperature, moisture (i.e. differences between control and treatment plots) and length of experiment. SE: Standard Error; CI.lb: confidence interval, low bound; CI.up: confidence interval, upper bound. Significance codes: 0.01 < P < 0.05 '*', 0.05 < P < 0.1 '†'.

Variable	Factor	Estimate	SE	Z value	P value	CI.lb	CI.ub
MBN	Intercept	-0.170	0.554	-0.307	0.759	-1.256	0.916
	Temp	0.663	0.386	1.72	0.085 †	-0.092	1.419
	Moisture	0.106	0.065	1.619	0.106	-0.022	0.234
	Temp:Length	-0.023	0.030	-0.747	0.455	-0.081	0.037
	Temp:Moisture	-0.080	0.041	-1.986	0.047 *	-0.160	-0.001
	Moisture:Length	0.003	0.002	1.212	0.225	-0.002	0.007
NH_4^+	Intercept	-0.620	0.435	-1.426	0.154	-1.471	0.232
	Temp	0.178	0.421	0.423	0.672	-0.646	1.002
	Moisture	0.007	0.047	0.149	0.882	-0.086	0.100
	Temp:Length	0.031	0.044	0.715	0.475	-0.054	0.116
	Temp:Moisture	-0.009	0.023	-0.373	0.709	-0.054	0.037
	Moisture:Length	0.002	0.004	0.431	0.667	-0.007	0.011
NO_3^-	Intercept	-0.042	0.454	-0.093	0.926	-0.931	0.847
	Temp	0.029	0.454	0.064	0.949	-0.861	0.919

	Moisture	0.176	0.163	1.077	0.282	-0.144	0.496
	Temp:Length	-0.005	0.064	-0.077	0.939	-0.130	0.120
	Temp:Moisture	-0.08	0.079	-1.009	0.313	-0.234	0.075
	Moisture:Length	-0.034	0.031	-1.105	0.269	-0.094	0.024
DON	Intercept	-0.560	0.376	-1.489	0.137	-1.298	0.178
	Temp	0.152	0.320	0.475	0.635	-0.475	0.779
	Moisture	-0.099	0.054	-1.830	0.067 †	-0.205	0.007
	Temp:Length	0.014	0.025	0.556	0.578	-0.036	0.064
	Temp:Moisture	0.054	0.031	1.779	0.075 †	-0.006	0.114
	Moisture:Length	-0.001	0.002	-0.312	0.755	-0.004	0.003
TN	Intercept	0.105	0.245	0.430	0.667	-0.375	0.586
	Temp	-0.079	0.203	-0.392	0.695	-0.476	0.318
	Moisture	-0.013	0.018	-0.751	0.453	-0.048	0.022
	Temp:Length	0.006	0.026	0.208	0.836	-0.046	0.057
	Temp:Moisture	0.007	0.018	0.396	0.692	-0.027	0.041
	Moisture:Length	-0.0002	0.001	-0.152	0.88	-0.002	0.002
Nmin	Intercept	0.377	3.122	0.121	0.904	-5.741	6.496
	Temp	-0.087	4.201	-0.021	0.983	-8.322	8.147
	Moisture	0.072	0.157	0.460	0.645	-0.236	0.381
	Temp:Length	0.002	0.428	0.004	0.997	-0.837	0.840
	Temp:Moisture	0.097	0.281	0.345	0.730	-0.454	0.649
	Moisture:Length	-0.031	0.055	-0.565	0.572	-0.139	0.077
Nfix	Intercept	0.223	1.004	0.222	0.824	-1.746	2.192

	Temp	0.148	1.005	0.147	0.883	-1.822	2.118
	Moisture	-0.859	0.436	-1.971	0.049 *	-1.713	-0.005
	Temp:Length	-0.026	0.037	-0.716	0.474	-0.098	0.046
	Temp:Moisture	0.753	0.391	1.924	0.054 †	-0.014	1.519
	Moisture:Length	0.011	0.005	2.062	0.039 *	0.001	0.022
Fungi	Intercept	0.129	2.177	0.059	0.953	-4.138	4.397
	Temp	2.392	3.038	0.787	0.431	-3.562	8.346
	Moisture	0.350	0.461	0.759	0.448	-0.553	1.254
	Temp:Length	-0.415	0.44	-0.943	0.346	-1.277	0.448
	Temp:Moisture	-0.693	0.772	-0.897	0.370	-2.207	0.821
	Moisture:Length	0.157	0.173	0.908	0.364	-0.182	0.495
Bacteria	Intercept	0.161	1.216	0.132	0.895	-2.223	2.545
	Temp	3.990	1.914	2.085	0.037 *	0.239	7.742
	Moisture	3.301	1.896	1.741	0.082 †	-0.415	7.016
	Temp:Length	-1.020	0.459	-2.223	0.026 *	-1.919	-0.121
	Temp:Moisture	-2.373	1.284	-1.848	0.065 †	-4.89	0.143
	Moisture:Length	-0.241	0.213	-1.129	0.259	-0.659	0.178
Roots	Intercept	0.543	0.707	0.768	0.443	-0.843	1.930
	Temp	-0.619	0.597	-1.036	0.300	-1.789	0.552
	Moisture	-0.077	0.070	-1.093	0.275	-0.214	0.061
	Temp:Length	0.039	0.053	0.731	0.465	-0.065	0.143
	Temp:Moisture	0.010	0.033	0.300	0.764	-0.055	0.075
	Moisture:Length	0.005	0.010	0.485	0.628	-0.015	0.025

References

Lamb, E. G., Han, S., Lanoil, B. D., Henry, G. H., Brummell, M. E., Banerjee, S., Siciliano, S. D. (2011). A High Arctic soil ecosystem resists long-term environmental manipulations. Global Change Biology, 17(10), 3187-3194.

Rossi, S., Bordeleau, A., Morin, H., Houle, D. (2013). The effects of N-enriched rain and warmer soil on the ectomycorrhizae of black spruce remain inconclusive in the short term. Annals of Forest Science, 70(8), 825-834.

Woodward, F. I., Lomas, M. R., Kelly, C. K. (2004). Global climate and the distribution of plant biomes. Philosophical Transactions of the Royal Society of London. Series B: Biological Sciences, 359(1450), 1465-1476.
