# Supplementary material for: Faster nitrogen cycling and more fungal and root biomass in cold ecosystems under experimental warming: a meta‐analysis
Source: Ecology. 2019 Dec 31;101(2):e02938. doi: 10.1002/ecy.2938 (PMC7027553; doi:10.1002/ecy.2938)
Supplement: Supplementary file 3 — MetadataS1 [file ECY-101-e02938-s003.pdf]

Alejandro Salazar, Kathrin Rousk, Ingibjörg S. Jónsdóttir, Jean-Philippe Bellenger, Ólafur S. Andrésson. 2019. Faster nitrogen cycling and more fungal and root biomass in cold ecosystems under experimental warming: a meta-analysis. *Ecology.*

Data S1

Biomass, enzymatic, genetic, biogeochemical and environmental data of control and experimentally warmed plots in the 100 datasets (94 studies) reviewed in this meta-analysis. Details of each experimental site (i.e. coordinates, MAT, MAP, soil depths, biome classification, averaged temperature and moisture difference between control and treatment plots, duration of warming experiment, and references) are also included.

Authors

Author Name: Alejandro Salazar Villegas
Affiliation: Faculty of Life and Environmental Sciences, University of Iceland.
Address: Sturlugata 7, 101 Reykjavík, Iceland.
Email: alejandro.salazar-villegas@fulbrightmail.org

Author Name: Kathrin Rousk
Affiliation: Department of Biology, Terrestrial Ecology Section, University of Copenhagen; Center for Permafrost (CENPERM), University of Copenhagen.
Address: Universitetsparken 15, 2100 Copenhagen, Denmark; Øster Voldgade 10, 1350 Copenhagen, Denmark.
Email: kathrin.rousk@bio.ku.dk

Author Name: Ingibjörg S. Jónsdóttir
Affiliation: Faculty of Life and Environmental Sciences, University of Iceland.
Address: Sturlugata 7, 101 Reykjavík, Iceland.
Email: isj@hi.is

Author Name: Jean-Philippe Bellenger
Affiliation: Centre Seve, Department of Chemistry, Faculty of Sciences, Universite de Sherbrooke.
Address: J1K2R1 QC, Canada.
Email: Jean-Philippe.Bellenger@usherbrooke.ca

Author Name: Ólafur S. Andrésson
Affiliation: Faculty of Life and Environmental Sciences, University of Iceland.

Address: Sturlugata 7, 101 Reykjavík, Iceland.
Email: osa@hi.is

File list

`raw_data.csv`

Description

Abbreviations

All headings contain the letter c – Control plots

All headings contain the letter w – Warmed plots

n (columns E and DS) – Number of replicates

se – standard error

sd – standard deviation

MicN – Microbial biomass N

DON – Dissolved Organic N

NH4 – Ammonium

NO3 – Nitrate

TSN – Total soil N

Nfix – N fixation

Nmin – N mineralization

Nnit – N nitrification

Namo – N ammonification

N2O – N_2O emissions

Ure – Urease activity

Pro – Protease activity

LAP – Leucine aminopeptidase (LAP) activity

NAG – N-acetylglucosaminidase (NAG) activity

PO – Phenoloxidase (PO) activity

POX – Peroxidase (POX) acitivity

Bac – Bacterial biomass

GramP – Gram positive

GramN – Gram negative

Act – Actinobacteria

Aci – Acidobacteria

Apro – α-proteobacteria

Fun – Fungal biomass

AMF – Arbuscular mycorrhiza

EM – Ectomycorrhizae

Arch – Archaea

BacF – Bacterivore

FunF – Fungivore

BamoA – Bacterial amoA

AamoA – Archaeal amoA

nosZ – nosZ

nifH – nifH

nirK – nirK

`nirS` – nirS

`root` – Root biomass

`root N` – Root N content

`ST` – Soil temperature

`SM` – Soil moisture

`pH` – Soil pH
